# Optical Diagnostics in the Gaseous Electronics Conference Reference Cell

**DOI:** 10.6028/jres.100.028

**Published:** 1995

**Authors:** G. A. Hebner, K. E. Greenberg

**Affiliations:** Sandia National Laboratories, Dept. 1128, MS 1423, Albuquerque, NM 87185; University of New Mexico, Department of Chemical and Nuclear Engineering, Albuquerque, NM 87131

**Keywords:** absorption, argon, discharge, electric field, Gaseous Electronics Conference Reference Cell, helium, laser-induced fluorescence, metastable, parallel plate, plasma processing, radio frequency

## Abstract

A number of laser-induced fluorescence and absorption spectroscopy studies have been conducted using Gaseous Electronics Conference Reference Cells. Laser-induced fluorescence has been used to measure hydrogen atom densities, to measure argon metastable spatial profiles, to determine the sheath electric field, and to infer the electron density and temperature. Absorption spectroscopy, using lamp sources and diode lasers, has been used to measure metastable atom densities in helium and argon discharges and fluorocarbon densities in silicon etching discharges. The experimental techniques and sample results of these investigations are reviewed.

## 1. Introduction

Plasma processes are used in a large number of applications ranging from production of high-density integrated circuits to waste remediation and lighting. Commonly, development of a commercial plasma process is achieved by empirical methods: all of the input parameters are varied until the desired output is obtained. Unfortunately, development and optimization of processes by empirical methods can be extremely costly and time consuming. The alternative to relying on empirical methods is to develop accurate plasma models (numerical simulations) having predictive capabilities that can be used to design and optimize systems and processes.

Due to the highly complex nature of processing plasmas, sophisticated computer codes are needed to predict plasma properties from first principles. Benchmarking plasma simulations against measured discharge parameters such as electron density, excited-state density, radical density, optical emission, and sheath electric field insures that the codes are computationally correct and that they include the important kinetic processes. One of the reasons for developing the Gaseous Electronics Conference (GEC) Reference Cell was to facilitate comparisons of experimental and theoretical data. By establishing a common geometry that could be used by both experimentalists and theoreticians, it was thought that a large data base of experimental results would be developed and used to verify the accuracy of various numerical simulations.

A variety of experimental measurements of plasma properties have been made using GEC Reference Cells. The experimental measurements were conducted to gain insight into plasma physics, to provide data that can be used for code validation and, in some cases, to demonstrate the use of diagnostics for process control. In this paper, we review some of the optical diagnostic techniques that have been applied in studies with Reference Cells as well as selected results from those studies.

To date, the optical diagnostics that have been applied in studies with the Reference Cell can be grouped into four categories: laser-induced fluorescence (LIF), absorption spectroscopy, optical emission spectroscopy, and dynamic laser light scattering. This paper will only address the laser-induced fluorescence and absorption measurements; optical emission [1] and dynamic laser light scattering [2] results are presented in separate review articles. In particular, we discuss LIF measurements of atomic hydrogen densities, argon metastable spatial distributions, sheath electric fields in helium, and electron density and temperature in helium. Absorption measurements of atomic metastable densities in helium and argon discharges and molecular species densities in silicon etching discharges are also reviewed.

## 2. Laser-Induced Fluorescence Measurements

Laser-induced fluorescence is a relatively non-perturbative technique that can be used for a variety of measurements in plasma systems. For example, using LIF it is possible to identify and monitor species [3–5], determine translational energies from measured Doppler profiles [6–8], and determine the sheath electric field from measured Stark shifts of energy levels [9–12]. LIF has been applied in studies with the Reference Cell to investigate plasma-surface interactions, to monitor densities and spatial distributions, and to measure sheath electric fields.

### 2.1 Plasma-Surface Interactions

Interactions between the plasma and surfaces in contact with the plasma (electrodes and chamber walls) can strongly affect discharge properties. It is important to understand how surface phenomena such as passivation, etching, species recycling, and secondary emission (ion and electron) influence chemistry and physics both in the sheath and in the volume of a discharge. Ganguly and Bletzinger have recently investigated the effects of various surface materials on pure H_2_ discharge properties in a Reference Cell [13]. In particular, they measured atomic hydrogen radial profiles above “split” electrodes made up of two different materials.

For their measurements, Ganguly and Bletzinger used a Reference Cell with the “standard” 2.54 cm interelectrode spacing. The Cell was driven asymmetrically; power was applied at 13.56 MHz to the upper aluminum showerhead electrode while the lower electrode and remainder of the chamber were grounded. The lower (grounded) electrode was a “split” electrode and was made up of aluminum and nickel or aluminum and copper.

Atomic hydrogen radial profiles were measured using two photon LIF. A Nd:YAG pumped dye laser generated 615 nm pulses that were frequency tripled in potassium dihydrogen phosphate (KDP) and β-barium borate (BBO) crystals to produce 300 µJ of laser light near 205 nm. The 205 nm output was used to excite atomic hydrogen from the 1*s* state to the 3*s* and 3*d* states. Fluorescence was observed at 656.3 nm using a photomultiplier tube and a narrow bandpass filter. The laser excitation was phase locked to the radio frequency (rf) plasma excitation and, by sampling the photomultiplier tube output with and without the laser excitation, it was possible to accurately subtract the plasma induced hydrogen alpha emission from the LIF signal. The radial profiles were obtained by simultaneously scanning the laser beam, photon collection optics, and photomultiplier tube. Corrections for vignetting near the electrodes were obtained from hydrogen LIF measurements of photodissociated C_2_H_2_.

**Fig. 1:**
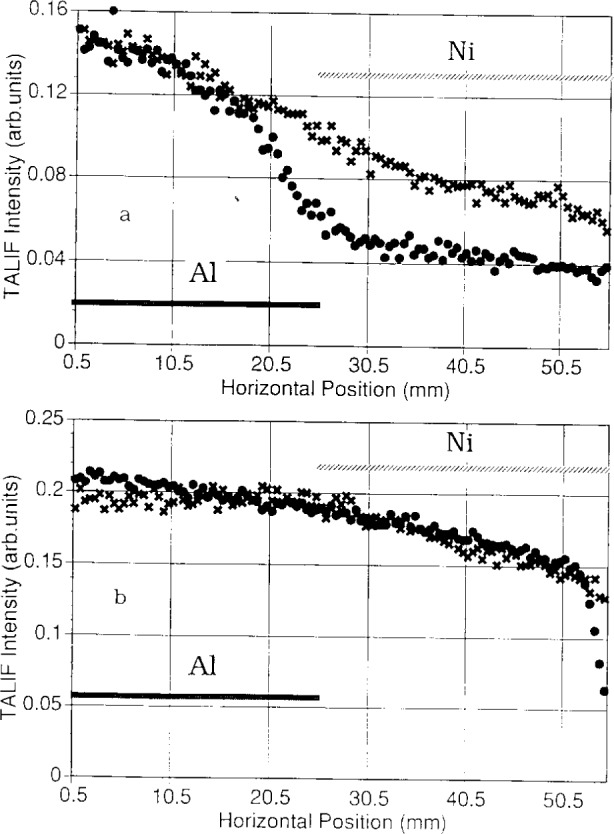
(a) Two-photon LIF scan parallel to the grounded bottom aluminum electrode (discharge vertical). Half of the aluminum electrode surface is covered by a thin nickel sheet. Horizontal location of the two electrode materials is indicated. The top electrode is connected to an rf generator via a matching network. The horizontal view is limited by the observation window. Pressure 133 Pa (1 Torr). Height of scan above the surface: • 3 mm, × 12 mm. (b) Same as (a), but after 10 h of operation (from Ref. [13]).

Figure 1 shows radial variations of the hydrogen LIF signals for a 133 Pa (1 Torr) hydrogen discharge with 35 W power into the plasma. The split lower electrode was composed of aluminum and nickel and the figure shows H atom densities measured 3 mm and 12 mm above the electrode. The data span roughly the central 5 cm of the electrode. The H-atom density profiles observed 3 mm above the electrode showed a strong radial variation and were asymmetric about the center of the electrode (the aluminum-nickel boundary). Ganguly and Bletzinger note that since the two body volume recombination rate for atomic hydrogen is small, the H-atom profile should be determined by diffusion, surface recombination loss, and the H-atom production process. They conclude that the asymmetric radial variation of the H-atom density is due to the difference between the surface recombination rates for atomic hydrogen on aluminum and nickel. Furthermore, they observe that the electrode material affects the H-atom density not just near the surface, but also in the volume, as evidenced by the asymmetric radial variation of the H-atom signal (albeit much smaller) at 12 mm above the electrode.

Figure 1b shows the LIF signals after the discharge has been operated for 10 h. The variation of the signal above the aluminum and nickel was less pronounced and led to the conclusion that the surface condition may have been modified. Secondary ion mass spectroscopy (SIMS) of the nickel sheet indicated that a thin layer of aluminum had coated the nickel. This result demonstrates that sputtering of different materials in a plasma reactor can significantly alter the plasma properties and, ultimately, the performance of the reactor.

### 2.2 Spatially Resolved Argon Metastable Density

Rare gas discharges are a natural starting point for developing and validating plasma numerical simulations, since a relatively complete set of cross section data is available, and discharge chemistry is minimized. Metastable species can be important in rare-gas discharges since they constitute a pool of relatively high energy atoms that can transfer energy and influence the discharge kinetics. Quantitative and qualitative measurements of metastable densities and spatial distributions are valuable for code validation.

McMillin and Zachariah have measured two-dimensional, time-averaged, argon (1*s*_5_) metastable spatial distributions using planar LIF imaging [14]. In their work, the output of a Nd:YAG pumped dye laser was frequency doubled in a KDP crystal to produce light at approximately 395 nm. The output laser beam was expanded vertically using a cylindrical lens telescope and was apertured to produce a 2×25 mm sheet of light. Laser energy and beam inhomogeneity were monitored by directing 5 % of the laser output into a static dye cell and recording the fluorescence with a charge coupled device (CCD) camera and frame grabber. The laser excited the 1*s*_5_ to 3*p*_2_ argon transition and fluorescence at 418 nm and 433 nm (3*p*_2_ to 1*s*_3_ and 1*s*_2_ transitions, respectively) was imaged at right angles to the illumination plane using a lens, bandpass filter, and an intensified CCD array. Fluorescence images were then corrected for plasma emission, CCD dark current, spectral response, flat field uniformity, laser energy distribution and collisional quenching changes of the fluorescence yield. The images were obtained by averaging 1000 laser pulses. Nominal axial and radial spatial resolution was 200 µm.

**Fig. 2:**
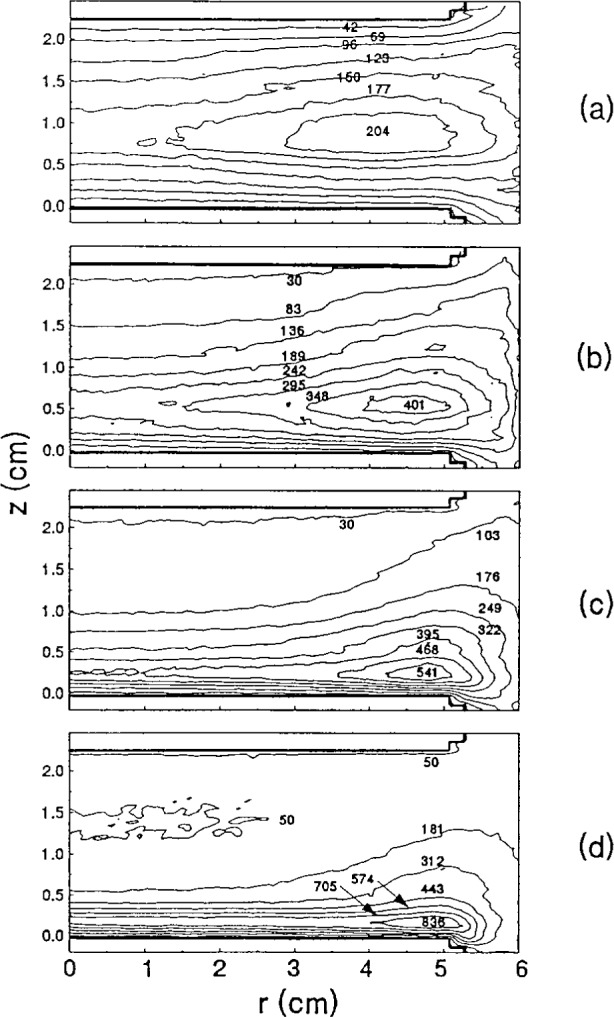
Contour plots of the argon 1*s*_5_ metastable relative density distributions in the GEC cell with an applied rf voltage of 200 V and pressures of (a) 13.3 Pa (0.1 Torr), (b) 33.3 Pa (0.25 Torr), (c) 66.7 Pa (0.5 Torr) and (d) 133.3 Pa (1.0 Torr) (from Ref. [14]).

Figure 2 shows contour plots of the relative argon 1*s*_5_ metastable density for a 200 V discharge operating at four different pressures: 13.3 Pa (0.1 Torr), 33.3 Pa (0.250 Torr), 66.7 Pa (0.5 Torr) and 133.3 Pa (1.0 Torr). The electrodes were driven asymmetrically with the lower electrode powered and the upper electrode grounded. Both the axial and radial metastable distributions depend strongly on the argon pressure. As the cell pressure was increased, the metastable axial distribution shifted from axially symmetric to strongly asymmetric while the radial distribution becomes strongly peaked at the edge of the electrode. McMillin and Zachariah attributed the change in the spatial distribution to the spatially dependent production and quenching rates of the metastables.

### 2.3 Sheath Electric Fields

The electric field in the sheath region of a gas discharge is a fundamental plasma parameter that is closely coupled to a number of other discharge parameters such as charge density, electron and ion energy distributions, and surface condition. Greenberg and Hebner have measured sheath electric fields in a Reference Cell using LIF to observe the structure of helium Rydberg Stark manifolds [15,16]. The measurements were made for both symmetrically and asymmetrically powered discharges. For symmetric operation, a push-pull drive maintained equal magnitude but opposite polarity voltages on the electrodes during the rf cycle. For asymmetric operation, the upper electrode was powered and the lower electrode was grounded. In both cases, the remainder of the Cell was maintained at ground potential.

The field measurements were made by using a laser to excite transitions from a helium metastable state to a Rydberg Stark manifold. When helium Rydberg states are excited by a laser, fluorescence emanating from many other levels not directly populated by the laser is observed. Measurements of laser induced fluorescence lifetimes indicates that collisional transfer can play an important role in population transfer [15,16]. By tuning the laser across the metastable-Rydberg transition and monitoring the LIF, it is possible to observe the Rydberg Stark structure. Electric fields were inferred by fitting the measured energy level splitting to calculated values.

For these experiments, the output of a 20 Hz Nd:YAG pumped dye laser was frequency doubled in a KDP crystal to produce 2 mJ to 4 mJ, 4 ns FWHM pulses near 320 nm. The laser output was synchronized with the rf cycle; pulse-to-pulse jitter was less than 1 ns and long term drift (80 min) was less than 2 ns. The 320 nm light was focused using a 0.5 m focal-length cylindrical lens and directed through the cell adjacent to the upper electrode. Throughout the discharge region, the beam extended from the electrode to approximately 5 mm below the electrode with a thickness of approximately 0.1 mm. The beam was polarized normal to the electrode surface. LIF was observed at 90 ° to the pump beam propagation direction using a gated, intensified, CCD camera. Typical spectra acquisition time was 20 s.

**Fig. 3:**
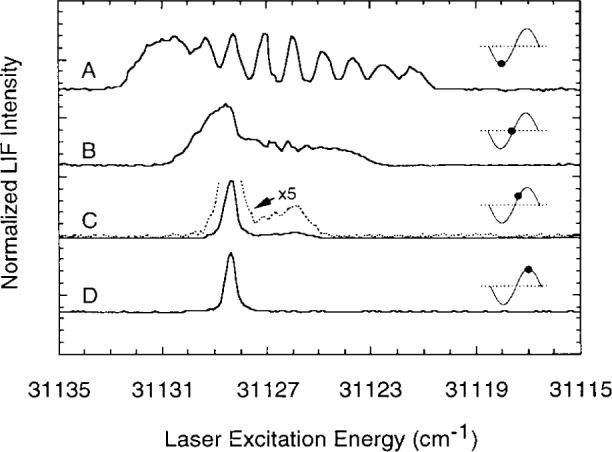
Normalized laser-excitation spectra recorded when the laser wavelength is tuned over the 1*s*11*p* ^1^P-1*s*2*s* ^1^S transition and fluorescence from the 1*s*4*d* ^3^D-1*s*2*p* ^3^P transition (447 nm) is monitored. The rf voltage was 200 V peak applied voltage at 66.7 Pa (0.5 Torr) (symmetric drive). The spectra A–D correspond to measurements at four different phases of the rf voltage at the electrode as specified by the markers on the sine wave insets. The fluorescence intensities have all been normalized. A five times intensity spectrum (dotted line) is also presented for case C to more clearly show the weak Stark structure (from Ref. [15]).

To measure the electric field, the laser was typically scanned over approximately a 20 cm^−1^ interval to excite the *n*=11 helium singlet Rydberg manifold. Figure 3 shows spectra observed 1 mm below the center (radius=0) of the upper driven electrode. The four different spectra (A–D) in the figure correspond to four different times during the rf cycle. The sheath electric field was expected to be greatest when the upper electrode had a maximum negative voltage applied to it; as expected, the Stark broadening was greatest for these conditions (spectrum A) with 8 of the 10 individual 1 levels in the pattern shown being clearly resolved (excitation of the 11 ^1^S level was not monitored in this experiment). From calculation of the Stark splitting of the *n*=11 singlet manifold, an electric field of 755 V/cm±25 V/cm was inferred. With no voltage applied to the electrodes (rf voltage zero crossing, spectrum B) a substantial sheath field of 500 V/cm±25 V/cm was still observed. A fairly large field must remain when the rf voltage passes through zero to keep the electrons in the plasma and maintain charge neutrality since the ions are essentially fixed in space on the time scale of the rf cycle. The field then dropped rapidly to about 100 V/cm±25 V/cm after another 1/8 rf cycle (spectrum C) and Stark broadening could not be detected at the positive peak of the applied rf voltage (spectrum D).

**Fig. 4:**
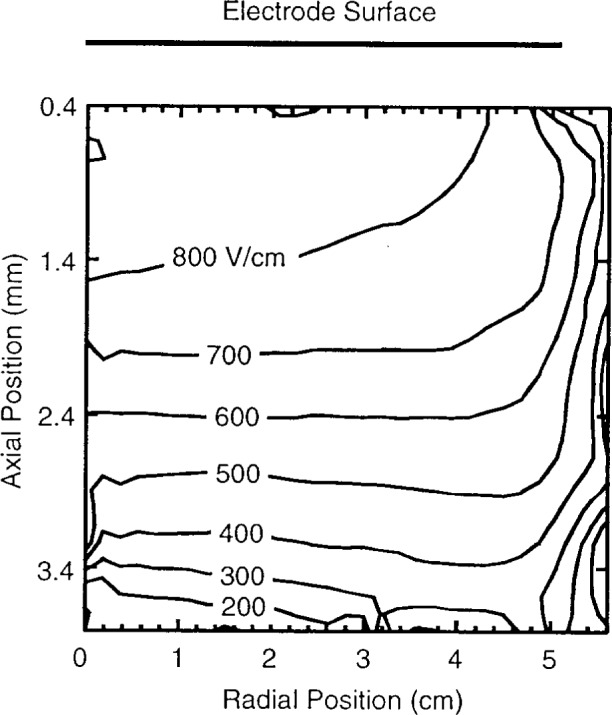
Constant electric field contours below the electrode for peak negative voltage. Discharge conditions: 66.7 Pa (0.5 Torr) helium, 200 V, push-pull drive, no dc bias (dc blocking capacitors shorted). The location of the electrode surface is shown by the line above the graph (from Ref [16]).

Figure 4 shows a two dimensional electric field map for a 66.7 Pa (0.5 Torr) discharge. The electrode was at peak negative voltage with 200 V applied using the push-pull (symmetric drive) configuration. The dc self bias was shorted to ground (i.e., the dc blocking capacitors were shorted). The location of the electrode surface is shown by the line above the graph. It should be noted that the axial dimension of the field map is millimeters while the radial dimension is centimeters. The contours of constant electric field are relatively flat over a large fraction of the electrode diameter, only rapidly decreasing within 5 mm of the electrode edge. The small decrease in electric field at 0.4 mm below the electrode and at *r*=5 cm is possibly due to fringing of the field to the ground shield around the electrode. The fact that the electric field was uniform over a large fraction of the electrode diameter suggests that 1-D calculations of sheath characteristics could be applicable in the area near the center of the discharge.

Hebner and Greenberg also examined the effect of the dc bias on the sheath electric fields. For a symmetrically driven discharge, they confirmed that the Reference Cell acts like a triode system and that the presence of a dc bias has little effect on the magnitude of the sheath field. With a push-pull (symmetric) drive, each electrode develops a dc bias with respect to the grounded Reference Cell chamber. However, there is no net dc bias across the electrodes and, consequently, the sheath fields measured with and without dc bias were similar.

### 2.4 Spatially Resolved Electron Density and Temperature

The absolute electron density and temperature are fundamental discharge parameters that control the properties of a plasma. Traditional methods of determining the electron density include microwave interferometry to measure a line integrated electron density [17] and Langmuir probes for point measurements [18]. Dzierzega, Musiol, and Roberts utilized a technique based upon LIF and electron collisional mixing to infer the electron density and temperature as a function of radial and axial position in helium discharges [19].

For their measurements, Dzierzega, Musiol, and Roberts used a Reference Cell with 10.2 cm diameter aluminum electrodes and a 2.5 cm interelectrode spacing. The lower electrode was powered while the upper electrode and chamber walls were grounded. The 637.4 nm output of a Nd:YAG pumped dye laser was frequency doubled to obtain approximately 1 mJ pulses at 318.7 nm. LIF was observed at 45 ° to the incident pump laser beam direction using a four mirror imaging system, monochromator and photomultiplier tube. Spatial resolution was 0.5 mm vertically and 5 mm horizontally.

To infer the electron density and temperature, several assumptions were made about the population of the excited states. These assumptions included that population transfer between one excited helium level and other higher levels was due to collisions with ground state atoms and thermal electrons, the concentration and temperature of the atoms was uniform, the atoms were near room temperature, and the energy difference between states with angular quantum numbers *L*>2 for *n*=4,5 was small compared to the electron temperature and comparable to the atom temperature. Based upon these assumptions, Dzierzega et al. grouped the 4 ^3^D and 4 ^3^F states into an effective 4*d*′ state and the 5 ^3^D, 5 ^3^F, and 5 ^3^G into an effective 5*d*′ state. Then they formulated a set of rate equations to describe the interactions of the populations of these levels after the 4 ^3^P state was populated by direct laser excitation from the 2 ^3^S metastable level. Based upon the assumptions, calculated rates, and lifetimes, the electron density and temperature were calculated from measurements of the fluorescence lifetimes of the 4 ^3^P-2 ^3^S (318.7 nm), 4 ^3^D-2 ^3^S (447.1 nm), and 5 ^3^D-2 ^3^P (402.6 nm) transitions.

**Fig. 5:**
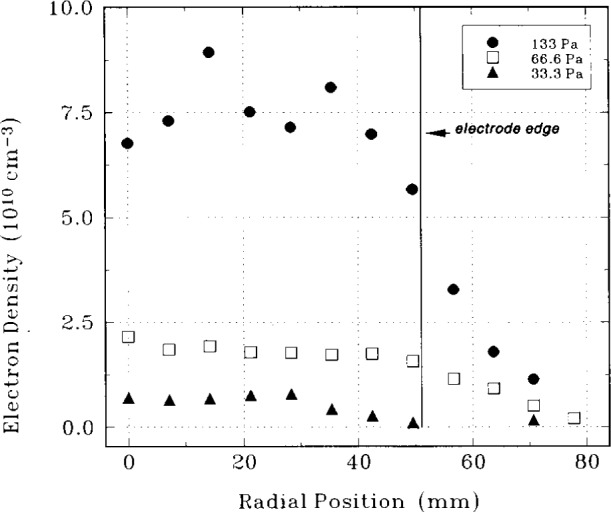
Electron density as a function of radial position for pressures of 133.3 Pa (circle), 66.7 Pa (square), and 33.3 Pa (triangle). The axial position was 12.5 mm above the lower electrode and the rf voltage was 200 V (from Ref. [19]).

The electron density as a function of radial position is shown in Fig. 5 for helium pressures of 33.3 Pa, 66.7 Pa, and 133.3 Pa. The peak to peak rf voltage was 200 V. For these conditions, the electron density was uniform over a large fraction of the electrode. The electron temperature was 0.6 eV±0.25 eV for all pressures and was insensitive to radial position and applied rf voltage. A comparison of the line averaged electron density 12.5 mm above the lower electrode with the microwave interferometer results of Greenberg and Hebner [17] shows agreement to within a factor of 4 for pressures of 66.7 Pa and 133.3 Pa and rf voltages of 200 V. However, the agreement for other operating conditions is not as good. Dzierzega and coworkers propose that the discrepancies could be due to spatial variation of the electron density; the LIF technique makes a point measurement while the microwave technique determines a line averaged density. Additional errors could be due to the uncertainties in the rate coefficients, and neglecting the influence of collisional transfer to other energy levels. A more complete comparison of all electron density measurements in the Reference Cell is presented in the review article by Overzet [18].

## 3. Absorption Measurements

Absorption spectroscopy has been used to measure metastable densities in argon and helium discharges and molecular species densities in etching discharges. If the absorption cross section is known or can be measured, this technique can provide absolute number densities. Absorption measurements have been performed using traditional lamp sources and diode lasers sources.

### 3.1 Absorption Measurements in Etching Discharges

In addition to providing information about the processes occurring in plasma systems, optical diagnostics also offer the possibility of non invasive real time process control in plasma processing reactors. Oh and coworkers have used diode laser absorption spectroscopy to monitor species concentrations in CF_4_/CHF_3_ etching plasmas in a Reference Cell [20]. They examined the use of absorption spectroscopy for process endpoint detection and for monitoring etch selectivity.

In these experiments, lead salt diode lasers were mounted on a temperature controlled copper block in a liquid nitrogen dewar. A resistive heater bonded to the copper block heated the lasers above liquid nitrogen temperatures for coarse wavelength tuning while high resolution tuning was provided by adjusting the current. The output of the lasers is collimated into a relatively wide (1 cm to 2 cm diameter) beam. Two lasers were mounted in the dewar to cover the wavelength range of 1200 cm^−1^ to 1300 cm^−1^ and 1040 cm^−1^ to 1120 cm^−1^. Using these two diodes, it was possible to excite CF_4_, CF_3_, CF_2_ (*ν*_1_ and *ν*_3_ bands), CF, and CF_2_O. High frequency laser wavelength modulation spectroscopy (WMS) was used to measure absorption in these experiments. The diode laser was tuned across an absorption profile by ramping the laser current. In addition, a small sinusoidal modulation was superimposed on the laser current. Phase sensitive detection was then used to observe signals at the sinusoidal modulation frequency or at a harmonic of the modulation frequency. Advantages of this technique, which is an extension of diode laser derivative spectroscopy, include minimization of laser and detector noise (typically 1/*f* limited) and increased sensitivity (single pass measurements are possible in the Reference Cell).

Using the diode laser system, Oh et al. measured the dissociation fraction of CF_4_ feed gas, neutral CF_2_ reaction intermediates, and CF_2_O etch products. They found, for example, that the CF_4_ fractional dissociation was approximately 25 % for a 66.7 Pa (0.5 Torr), CF_4_/CHF_3_ (0.87/0.13) discharge operating at 700 V. They calculated that the line integrated CF_2_ density through the center of the discharge was relatively large in the Cell, ranging from 5×10^12^ cm^−3^ to 3×10^13^ cm^−3^ for a variety of etching conditions. In addition, their data suggest that monitoring of the CF_2_ density may be useful for optimizing etch selectivity.

**Fig. 6:**
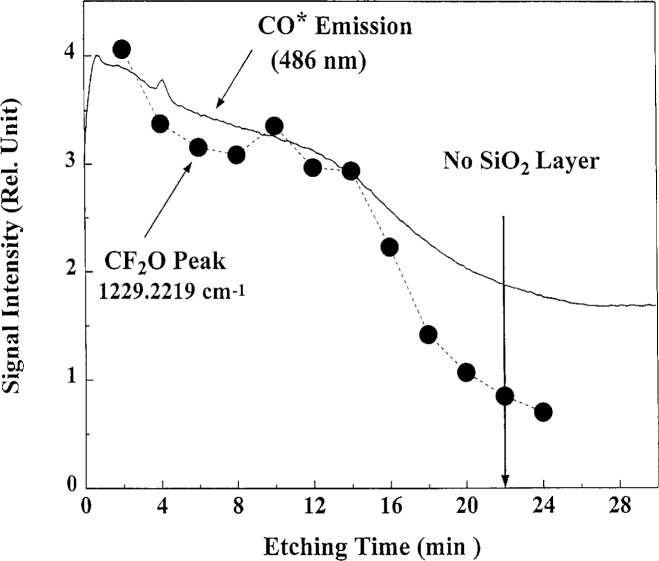
Comparison of the time dependence of CO* emission (486 nm) and CF_2_O absorption during etching of an SiO_2_ wafer. The etching conditions are 4.8 % CHF_3_/95.2 % CF_4_, 66.7 Pa (0.5 Torr) and 700 V peak to peak rf voltage (from Ref. [20]).

Absorption spectroscopy of CF_2_O was evaluated as a possible etch end-point detection technique. The absorption of CF_2_O (at 1229 cm^−1^) and optical emission of CO^*^ are shown in Fig. 6 as a function of time during etching of a blanket oxidized silicon wafer. Both the CF_2_O and CO* signals followed the same trend. The authors suggest that CF_2_O absorption could be used as an end-point detection sensor in situations were optical emission fails, such as when changes in an etching recipe change the optical emission characteristics of the plasma. The absence of a sharp decrease in the absorption and optical emission was attributed to poor spatial etch uniformity of the Reference Cell under these conditions.

### 3.2 Argon Metastable Density

In addition to the LIF measurements, spatial distributions of argon metastable concentrations have also been measured in a Reference Cell using absorption spectroscopy. For these experiments, Augustyniak, Filiminov, and Borysow used single mode diode lasers operating at 801.5 nm and 750.4 nm to measure populations of the 1*s*_5_ and 1*s*_2_ levels [21]. The diode laser was tuned across the spectral lineshapes of the absorbing transition. Line integrated absorption was measured as a function of both axial and radial position with a spatial resolution of 1 mm and then Abel inverted to obtain radial density distributions. At low rf powers, densities of the 1*s*_5_ metastables were obtained from absorption at line center. At higher rf powers, however, the plasma was optically thick and, as a result, the density was inferred from an analysis of the lineshape in the wings of the absorption. Under these conditions (high rf powers), the absolute center wavelength of the argon transition was obtained from an external reference discharge.

**Fig. 7:**
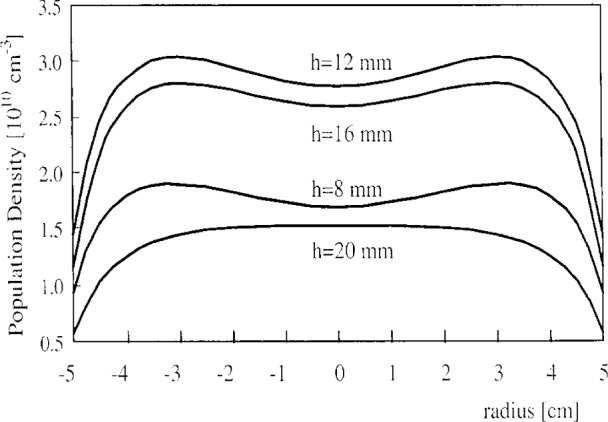
Radial distribution of the absolute number density of 1*s*_5_ argon atoms obtained from integrated absorption profiles. The number by each curve denotes the distance from the bottom (powered) electrode. The measurements were taken at a nominal rf power of 1 W and pressure 20 Pa (0.15 Torr) (from Ref. [21]).

The Reference Cell was driven asymmetrically in this work, with the bottom electrode powered and the top electrode grounded. The interelectrode spacing was 25.4 mm. Radial line-integrated 1*s*_5_ metastable profiles are shown in Fig. 7 for several heights above the bottom electrode. The Cell pressure was 20 Pa (0.15 Torr) and the rf power was 1 W. As shown, the 1*s*_5_ density was maximum near the midplane (12.7 mm) between the electrodes. Close to the grounded upper electrode, the density was relatively flat over the central portion of the electrode and decreased monotonically moving away radially from the center of the Cell. At the other axial positions, the metastable density was peaked near the electrode edges. This is similar to the LIF results of McMillin and Zachariah [14] who also observed argon metastables densities that peaked radially near the edge of the electrode (*r*=5 cm) for higher pressures and rf powers.

### 3.3 Helium Metastable Density

Greenberg and Hebner used absorption spectroscopy to measure the helium singlet and triplet metastable densities throughout the volume of discharges in a Reference Cell [17,22]. Light from a helium Geisler discharge tube was expanded and collimated using 15.2 cm diameter lens. The collimated light was spatially filtered and directed through the Reference Cell such that it illuminated the entire volume between the electrodes, extending radially from the center of the cell to the edge of their optical window. The light exiting the Cell was focused through a 0.5 mm diameter aperture and imaged through a bandpass filter onto a CCD camera. Light at 388.9 nm (2 ^3^S-3 ^3^P) was used to measure the 2 ^3^S helium metastable density while absorption at 501.4 nm (2 ^1^S-3 ^1^P) was used to measure the 2 ^1^S metastable density. The spatial resolution was 0.5 mm. Measured absorption was converted to a line integrated density and then Abel transformed to infer the radial metastable density as a function of interelectrode position.

**Fig. 8:**
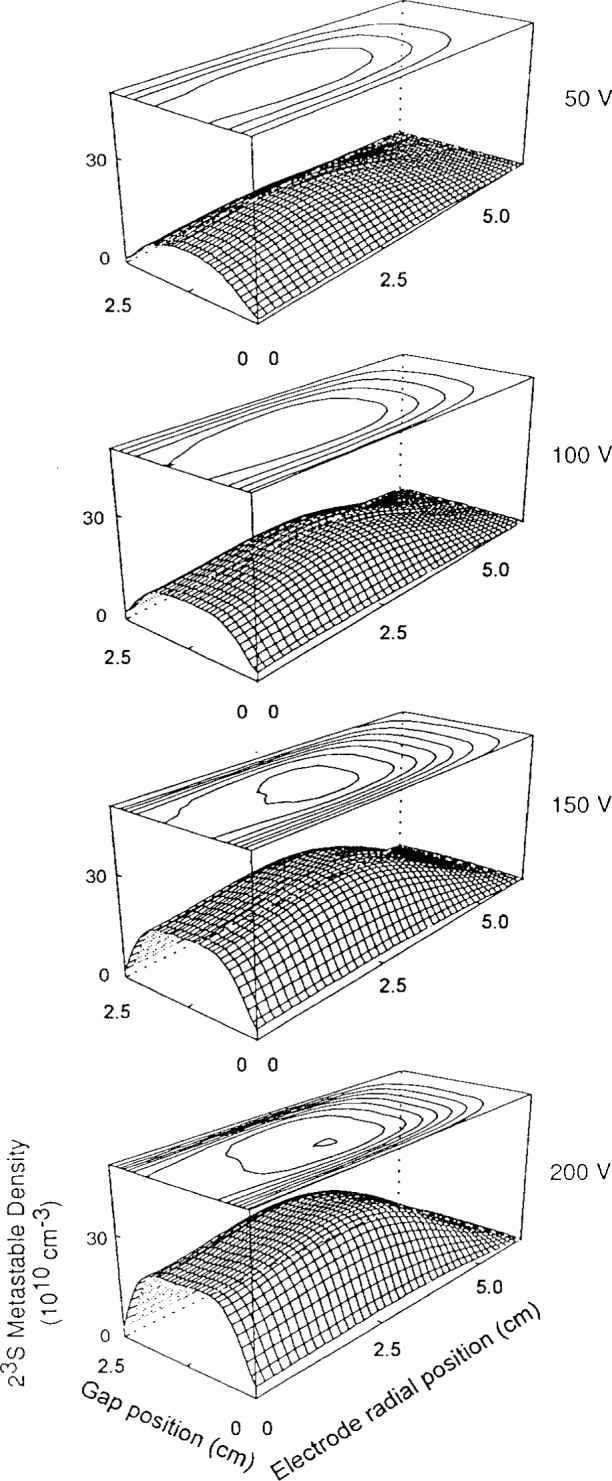
Helium triplet metastable density profiles as a function of voltage at 133 Pa (1 Torr). The surface mesh plots represent the spatial metastable density distribution and the line plots show contours on constant density. The minimum contour for the 50 V, 100 V, and 150 V cases corresponds to a density of 5×10^10^ cm^−3^ while the minimum contour for the 200 V case corresponds to a density of 8×10^10^ cm^−3^. In all cases, the minimum contour is located at the largest electrode radial position and the contour spacing is 3×10^10^ cm^−3^ (from Ref. [17]).

**Fig. 9:**
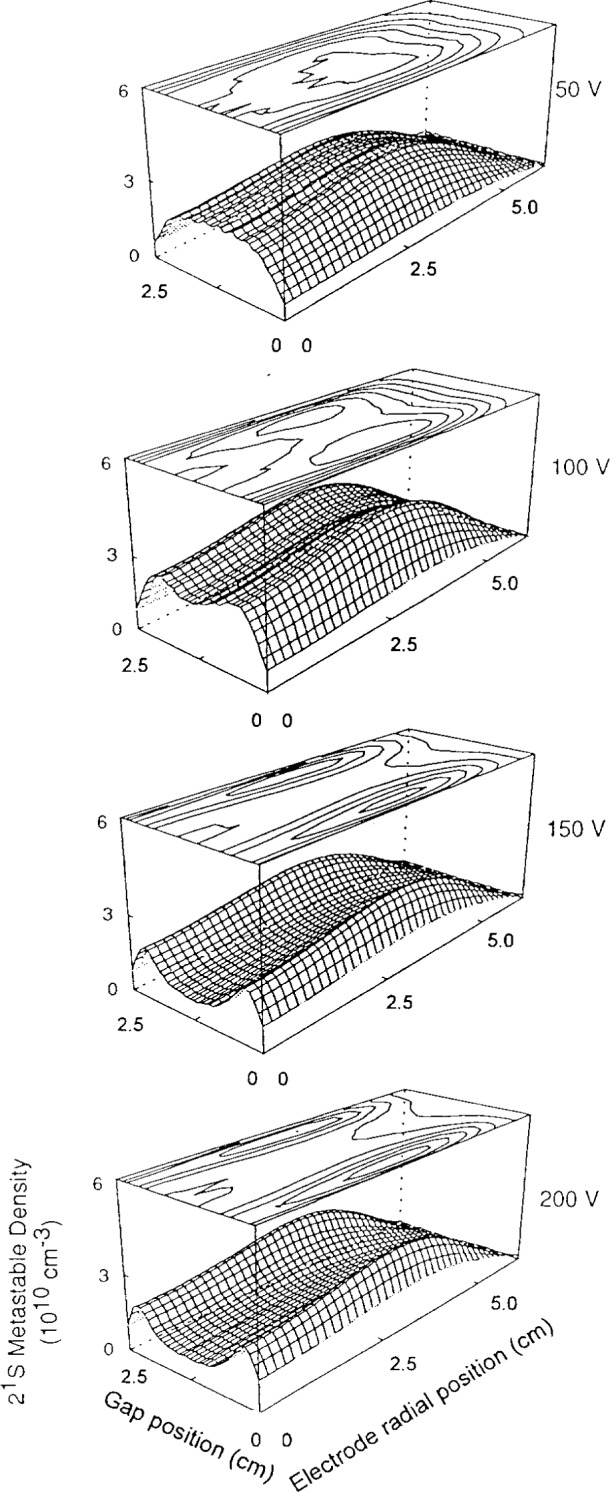
Helium singlet metastable density profiles as a function of voltage at 133 Pa (1 Torr). The surface mesh plots represent the spatial metastable density distribution and the line plots show contours on constant density. The minimum contour for the 50 V, 150 V, and 200 V cases corresponds to a density of 8×10^9^ cm^−3^ while the minimum contour for the 100 V case corresponds to a density of 1.2×10^10^ cm^−3^. In all cases, the minimum contour is located at the largest electrode radial position and the contour spacing is 4×10^9^ cm^−3^ (from Ref. [17]).

Helium triplet and singlet metastable densities are shown in Figs. 8 and 9 as a function of position between the electrodes for several applied rf potentials at 133.3 Pa (1.0 Torr). In these figures, the electrode radial position denotes position moving radially from the center of the cell (radius=0) toward the chamber walls; the electrode edge is at 5 cm. The gap position varies from 0 cm to 2.5 cm (the electrode separation) with 0 cm being the bottom electrode position and 2.5 cm being the position of the top electrode. The Abel inverted profiles all indicated that the metastable density peaked radially away from the center of the discharge chamber, the density being typically 15 % lower in the center of the discharge relative to the peak. The profiles indicate that the radial metastable densities were relatively constant over a large fraction (3/4) of the electrode diameter. This small variation over a large fraction of the electrodes suggests that one-dimensional models might be suitable for describing the central portion of the discharge. In contrast to the previously discussed argon metastable measurements, the rf electrodes for the helium metastable measurements were driven symmetrically. This directly leads to axially symmetric metastable density distributions.

As shown in Fig. 9, the singlet density was strongly depleted in the center of the 133.3 Pa (1.0 Torr) discharges at higher voltages. This depletion was not observed in the 66.7 Pa (0.5 Torr) discharges. The decrease in singlet density is probably due to electron collisional induced transfer between the single and triplet metastable states. Since the energy difference between the two metastable states is 0.78 eV and the cross section for collisional transfer is relatively large (9×10^−16^ cm^2^ for 1 eV electrons) the steady state metastable density will depend strongly on the characteristic electron energy. The depletion of the singlet density at higher voltages, observed by Greenberg and Hebner, would be consistent with a drop in the characteristic energy and could indicate that the Reference Cell operates in the γ mode at higher voltages and pressures [23].

## 4. Conclusions

Laser-induced fluorescence and absorption spectroscopy have been used to measure various species and electric fields in GEC Reference Cells. The results of these experimental studies have several implications for operation and modeling of discharges in a Reference Cell. First, measurements of hydrogen atom densities in pure H_2_ discharges have shown that electrode materials and sputtering can affect chemistry and physics both in the sheath region and in the volume of a discharge. This suggests that Reference Cells having different electrode materials (e.g., stainless steel versus aluminum electrodes) may display different discharge characteristics under certain conditions. Second, measurements of electric field strengths and electron density have shown that sheath fields and electron density are spatially uniform over a large fraction of the electrode radius. In addition, the radial variation of the metastable densities were also found to be constant over the central region of the discharge. This suggests that one-dimensional discharge models might be suitable for describing the central portion of the discharge under some conditions. Third, both the metastable and electric field measurements have shown the existence of electrode-edge effects. In particular, measurements of both helium and argon metastables in rare-gas discharges have shown that the metastable density peaks close to the electrode edge. Consequently, two- or three-dimensional models will be needed to completely describe discharges in the Reference Cell. Fourth, measurements of helium metastable densities indicate that the Reference Cell may operate in the *γ*-mode for higher voltage and pressure discharges through helium. Fifth, electric field measurements have shown that the sheath field strengths are independent of dc bias when the Cell is powered symmetrically. This suggests that it could be possible to obtain meaningful comparisons of experimental and theoretical data even if the numerical simulation does not include a dc bias.

Due to excellent optical access, the Reference Cell has proven to be a good medium for investigations using laser-induced fluorescence and absorption spectroscopy. To date, a significant amount of data has been obtained on rare-gas discharges. It is anticipated that future studies will concentrate on more complex gas systems of interest to commercial industry.
